# The paucity of morality in everyday talk

**DOI:** 10.1038/s41598-023-32711-4

**Published:** 2023-04-12

**Authors:** Mohammad Atari, Matthias R. Mehl, Jesse Graham, John M. Doris, Norbert Schwarz, Aida Mostafazadeh Davani, Ali Omrani, Brendan Kennedy, Elaine Gonzalez, Nikki Jafarzadeh, Alyzeh Hussain, Arineh Mirinjian, Annabelle Madden, Rhea Bhatia, Alexander Burch, Allison Harlan, David A. Sbarra, Charles L. Raison, Suzanne A. Moseley, Angelina J. Polsinelli, Morteza Dehghani

**Affiliations:** 1 Department of Psychology, University of Southern California, Los Angeles, USA; 2 Department of Psychology, University of Arizona, Tucson, USA; 3 Department of Management, David Eccles School of Business, University of Utah, Salt Lake City, USA; 4 Dyson School of Applied Economics and Management, Johnson College of Business, Cornell University, Ithaca, USA; 5 Sage School of Philosophy, Cornell University, Ithaca, USA; 6 Marshall School of Business, University of Southern California, Los Angeles, USA; 7 Department of Computer Science, University of Southern California, Los Angeles, USA; 8 Brain and Creativity Institute, University of Southern California, Los Angeles, USA; 9 Department of Psychiatry, University of Wisconsin-Madison, Madison, USA; 10 Minnesota Epilepsy Group, Roseville, USA; 11 Department of Neurology, Indiana University School of Medicine, Indianapolis, USA; 12 Department of Human Evolutionary Biology, Harvard University, 11 Divinity Ave, Cambridge, MA 02138 USA

**Keywords:** Psychology, Human behaviour

## Abstract

Given its centrality in scholarly and popular discourse, morality should be expected to figure prominently in everyday talk. We test this expectation by examining the frequency of moral content in three contexts, using three methods: (a) Participants’ subjective frequency estimates (*N* = 581); (b) Human content analysis of unobtrusively recorded in-person interactions (*N* = 542 participants; *n* = 50,961 observations); and (c) Computational content analysis of Facebook posts (*N* = 3822 participants; *n* = 111,886 observations). In their self-reports, participants estimated that 21.5% of their interactions touched on morality (Study 1), but objectively, only 4.7% of recorded conversational samples (Study 2) and 2.2% of Facebook posts (Study 3) contained moral content. Collectively, these findings suggest that morality may be far less prominent in everyday life than scholarly and popular discourse, and laypeople, presume.

## Introduction

In religious and philosophical traditions, morality is held to have a singular importance; where moral considerations conflict with other considerations, many writers have insisted that moral considerations should always be “overriding”^1,2^. *Nothing*, the gospel of Mark (8:36) tells us, can make up for a moral failing: “For what shall it profit a man, if he shall gain the whole world, and lose his own soul?” Everyday moral thought, while perhaps less conspicuously demanding, seems to evince a similar conviction: moral lapses can appear unforgivable in ways that lapses in other domains, like the culinary and sartorial, are not.

This impression is substantiated by a range of empirical work. In cross-cultural research, for example, moral values have been found to reside at the top of “value hierarchies” in cultures around the world^3^, and studies of person perception repeatedly find that assessments of a person’s moral characteristics play a central, even dominant, role in impression formation^4,5^. Moreover, self-perceptions exhibit a “moralistic bias,” where people have inflated assessments of their own moral probity^6^. The need to perceive oneself as moral is a widespread psychological need, and feelings of being moral may help people identify times when life is going well^7^. Moral character traits are among the most important determinants of the overall impressions that people form^8,9^. People desire to be more moral^10^ even though they tend to fall into the illusion of moral superiority^11^, which makes them perceive themselves as more moral than others, in line with the better-than-average effect^12,13^. Finally, it has been argued that the self is “essentially moral,” in that people reliably judge moral attributes to be the most important contributors to personal identity^14,15^. These empirical findings suggest that morality is not specific to one or even a few domains of everyday life (e.g., vaccination^16^, online political discussions^17^); rather, morality plays an important role in a variety of socio-cognitive domains and interpersonal contexts.

If morality has such singular *importance*, it should be something that people *frequently* think about, talk about, and act on. However, this plausible supposition is largely untested. A notable exception is a study by Hofmann and colleagues^18^, who used experience sampling to estimate how frequently their participants committed, were the target of, witnessed, or learned about a “moral” or “immoral” event. Consistent with the prominence-of-morality hypothesis, their participants reported, in repeated questioning over several days, morally relevant content in 28.9% of the episodes (15.3% moral; 13.6% immoral), suggesting that moral concerns are, as Hofmann et al. conclude, “frequent and manifold” (p. 1340). This methodology can increase participants’ attention to moral issues, potentially enhancing their correct detection and reporting^19^; however, it can also inflate reporting through increased accessibility, confirmatory search, and demand characteristics^20,21^, perhaps compounded by social desirability effects^22,23^.

Since Hofmann and colleagues’ germinal work was published in 2014, a range of computational and ecologically-valid tools have been further developed to analyze morally relevant information from large-scale online data (e.g., Hoover et al.^24^) and daily social behavior (e.g., Bollich et al.^25^). These methods provide the opportunity to identify and describe moral phenomena as they appear in natural language throughout everyday life.

Verbal behavior is not, of course, the totality of human behavior: people think about things they don’t talk about, and they do things without talking about them. Nevertheless, it is plausibly surmised that the frequency with which people talk about a topic in their everyday lives is a meaningful indicator of the relevance of the topic to everyday life and/or the importance people place on it^26^: that people seem to talk about food and relationships more than they talk about fluctuations in the price of shoelaces is surely not accidental.

Computational and ecologically-valid methods provide the opportunity to identify and describe moral phenomena as they appear in natural language throughout everyday life, in both face-to-face and online interactions (e.g., Bollich et al.^25^; Kennedy et al.^27^). Here, we test the prominence of morality in everyday talk using these methods. To address whether self-reports inflate the frequency of moral concerns relative to observational data, we asked participants in Study 1 to estimate how often they talk about moral issues in daily conversations. We compare these estimates to recordings of daily conversations, collected with an ecological behavior-observation method, the Electronically Activated Recorder (EAR)^21,28^, a portable audio recorder that unobtrusively records samples of ambient sounds as participants go about their days (Study 2). Complementing the analysis of daily conversations, we also extracted moral content in a large number of Facebook status updates (Study 3). In addition to investigating the *frequency* of moral content, all studies analyzed the *composition* of moral content, using the typology of moral concerns proposed by Moral Foundations Theory (MFT): care/harm, fairness/cheating, loyalty/betrayal, authority/subversion, and purity/degradation^29,30^. Importantly, MFT is a *descriptive* (rather than *normative*) theory of human morality, rooted in cultural psychology and evolutionary theory. We emphasize that our use of the term “moral” is descriptive, not normative; that is, we are not trying to categorize morally “right” or “good” linguistic behavior. MFT simply proposes a typology of moral values that are important aspects of human social life across cultures. This pluralistic approach is both necessary and pragmatic for the current descriptive research. As such, all our references to “moral” in this work are descriptive.

## Study 1

In Study 1, participants estimated how often they talk about moral issues in daily talk as they go about their ordinary lives. Our purpose was to gauge people’s estimation of the prominence of morality in daily conversations. We do so in two samples and with different questions to ensure that people’s estimation is robust and not unique to a particular way of asking (i.e., across different demand characteristics).

### Methods

#### Participants

All study protocols were approved by our Institutional Review Board, and were carried out in accordance with relevant guidelines and regulations. An informed consent was obtained from all participants. We recruited Sample 1A from Amazon Mechanical Turk. We aimed for a sample of 400 participants; of these, 378 fully completed the survey. After excluding participants who failed either of two attention checks, the final sample was reduced to 354 participants. The average age was 40.3 years (min = 18, max = 78, *SD* = 14.1 years). The majority of participants were White or European American (74.9%), followed by Black or African American (8.5%); 208 participants (58.8%) identified as woman, 145 participants (41.0%) identified as man, and one participant identified as non-binary. Sample 1B was also recruited from Amazon Mechanical Turk. We aimed to recruit 200 participants, but in order to account for potential exclusions based on an attention check we recruited 234 participants. After excluding participants who failed an attention check, a total of 227 participants remained. The average age in Sample 1B was 39.3 years (min = 19, max = 80, *SD* = 13.6 years). The majority of participants were White or European American (78.0%), followed by Asian or Asian American (8.4%) and Black or African American (7.5%). In terms of gender, 122 participants (53.7%) identified as woman, 102 participants (44.9%) identified as man, and three participants identified as non-binary.

#### Measures

In Sample 1A, we asked participants “What percent of your daily conversations touch on aspects of morality?” The participants were able to type any numeric response between 0 and 100. As a robustness check, we also framed our question in a more intuitive way as “Out of 100 conversations on an average day, how many touch on morality?” Then we asked 5 questions following the definitions of each moral foundation (again on a 0–100 scale): (1) “Within your conversations that touch on morality, what percent of your daily conversations touch on aspects of care and protecting individuals from harm?”; (2) “Within your conversations that touch on morality, what percent of your daily conversations touch on aspects of cooperation, reciprocity, and cheating?”; (3) “Within your conversations that touch on morality, what percent of your daily conversations touch on aspects of loyalty to others, self-sacrifice, and patriotism?”; (4) “Within your conversations that touch on morality, what percent of your daily conversations touch on aspects of respecting authorities and traditions?”; (5) “Within your conversations that touch on morality, what percent of your daily conversations touch on aspects of maintaining physical and spiritual purity, and preventing degradation?”.

We also asked participants about the perceived prevalence of everyday topics, along with “morality” included as one conversational topic among the others. Specifically, we asked “What percent of your daily conversations/interactions is about the following topics?” Participants were instructed to choose a percent on a slider. This slider was capped at 50% to avoid unrealistically high responses. These 15 topics (entertainment, relationships, school or education, personal goals, technology, food, health, home chores, fashion, money, job, morality, sex, sports, and politics) were presented in randomized order. At the end of the survey, we asked demographic questions.

In Sample 1B, we aimed to replicate benchmarking statistics using a more intuitive and visual response option. We showed participants a pie chart with 20 color-separated slices and asked them to “Suppose this pie depicts all the conversations you had yesterday.” Next, they were asked to report on the 15 topics of conversations from Sample 1A. For example, the question about “personal goals” was “Now consider all the conversations in which you talked about personal goals. How many slices would that share of the pie be?” Each slice was recoded to be worth 5%. All questions were presented in randomized order.

### Results

When asked, “What percent of your daily conversations touch on aspects of morality?”, Sample 1A participants estimated that 25.2% (*Md* = 20.0%) of their daily conversations do so. When the question was framed as “Out of 100 conversations on an average day, how many touch on morality?”, the results were similar (*M* = 24.3%, *Md* = 15.0%). In terms of the composition of moral concerns, care (*M* = 33.5%, *Md* = 25.0%) and fairness (*M* = 20.5%, *Md* = 10.0%) were considered the most frequent moral topics, followed by loyalty (*M* = 17.8%, *Md* = 10.0%), authority (*M* = 16.5%, *Md* = 8.5%), and purity (*M* = 15.5%, *Md* = 5.0%).

**Figure 1:**
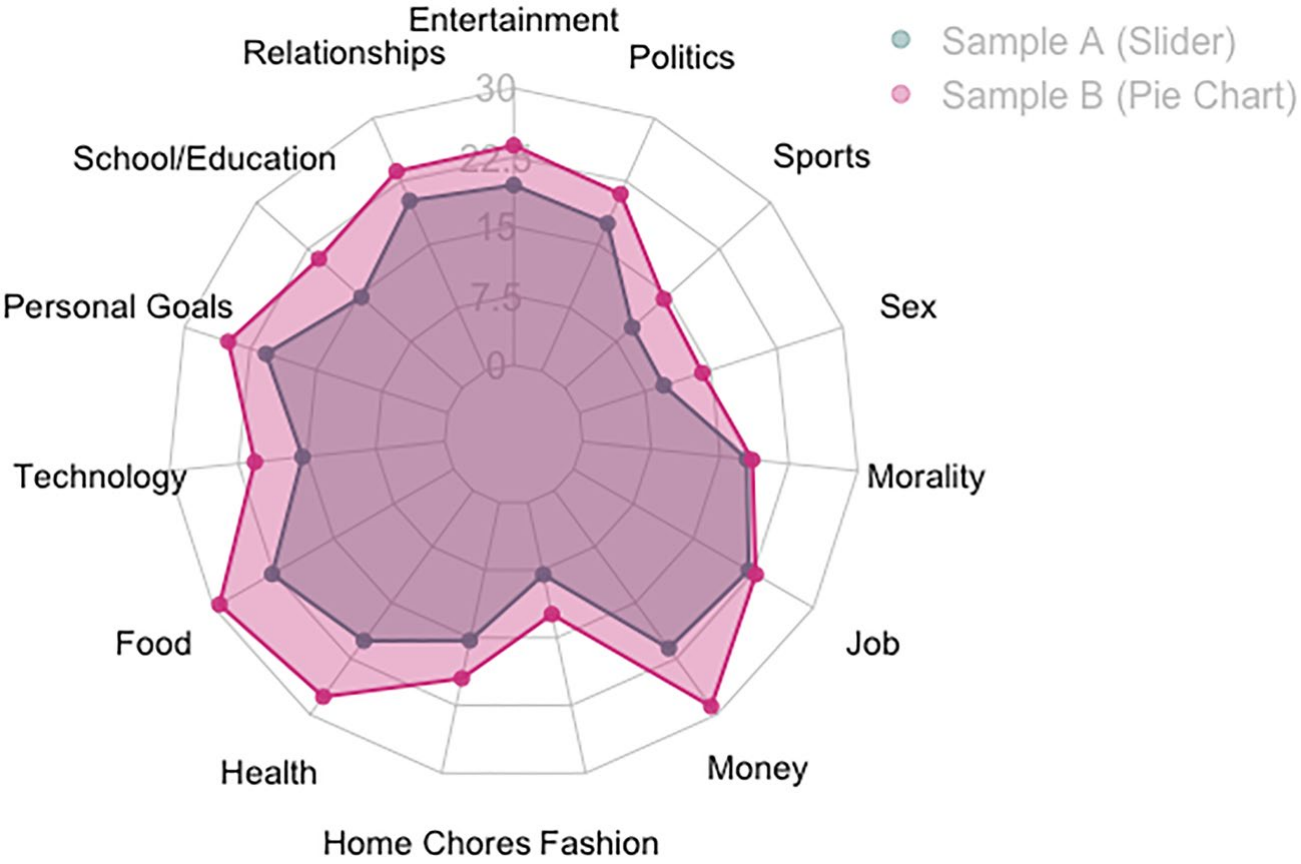
Participants’ estimation of frequency of different topics (Study 1).

We also benchmarked these frequencies against estimates for 15 other topics. In Sample 1A, participants indicated that they spent most time talking about food (*M* = 22.7%, *Md* = 20.0%) and career (*M* = 21.0%, *Md* = 20.0%) and the least time talking about fashion (*M* = 8.0%, *Md* = 2.0%) (see Supplementary Materials for comparisons and inferential statistics). Morality was perceived to be a fairly frequent topic, at an average of 17.9% (*Md* = 14.0%). We replicated these results in Sample 1B, using pie charts as a different response option with a sensitivity of 5% increments (see Supplementary Materials for full statistics). All benchmarking results are visually shown in Fig. 1.

These self-reports support the intuition that, from participants’ point of view, moral talk is “frequent and manifold”^18^ in everyday life. Our participants’ subjective *frequency* estimates, at around 21.5% (averaged across 4 assessments), are similar to Hofmann and colleagues’ self-reports of moral experiences, 28.9%. The similarity extends to the *composition* of moral concerns: in both studies, care was the most frequently reported moral concern, followed, in descending order, by fairness, loyalty, authority, and purity. Does the perceived prominence of moral concerns in everyday talk persist when morality is passively and objectively observed in everyday verbal interactions?

## Study 2

In this study, we aimed to assess the “observed” frequency of moral language. To examine the objective frequency of moral language in everyday conversations, we used the Electronically Activated Recorder (EAR; Mehl^21^), a smartphone application that collects a representative sample of daily conversations by intermittently, from morning to night, several times per hour, recording short ambient audio sound bites in participants’ sonic environment.

### Methods

Our annotation dataset consists of the spoken daily language of four groups of participants, captured using the EAR^21^. Implemented as a smart phone app, the EAR is designed to randomly record brief snippets of ambient sounds, including human conversations. Our sample consisted of 542 participants in the U.S., coming from four different samples and diverse backgrounds. Sample 2A consists of language data recorded for 208 medically healthy adults (65.37% female; *Min*_*age*_ = 25; *Max*_*age*_ = 55; *M*_age_ = 33.65) living in Atlanta, GA. Participants wore the EAR for one weekend before and after the eight-week meditation intervention, conducted by the Emory University Center for Health and Well-Being^31^. Sample 2B includes female breast cancer patients (with a primary diagnosis of Stage I, II, or III) and their cohabitating partners who were recruited from the Arizona Cancer Center (University of Arizona, Tucson) during regular visits to an oncologist (*N* = 105; 58.10% female; *Min*_*age*_ = 24; *Max*_*age*_ = 94; *M*_age_ = 57.61)^32^. Both members of the couple wore the EAR for one weekend. Sample 2C consists of 122 adults living in Tucson, AZ who had recently separated from their marital partners (71.31% female; *Min*_*age*_ = 24; *Max*_*age*_ = 65; *M*_age_ = 43.84)^33^. They wore the EAR for three weekends spanning four months. Finally, Sample 2D consists of 107 older adults living in Tucson, AZ (54.21% female; *Min*_*age*_ = 65; *Max*_*age*_ = 90; *M*_age_ = 76.04)^34^. They wore the EAR for five days, which included both weekday and weekend days. Notably, these four subsamples were selected through convenience sampling and were used because these were the samples that we had access to at the time of this research. Overall, we had 50,961 transcribed observations.

For all four sets of samples, all EAR sound files were transcribed, and then were considered for annotation, such that each annotation item includes the transcribed language collected during an activation period of the app for an individual. Transcripts with fewer than three words were not considered during annotation. See Table 1 for examples of transcribed snippets.

**Table 1:** Examples from the EAR (Study 2) and facebook (Study 3) datasets.

	EAR (Study 2)	Facebook (Study 3)
Care	*I love you too sweet pea. I love you okay*	*Penn puts it perfectly people need help who are we if we don't help people who need help*
Harm	*I mean the only thing that wouldn’t happen is if there were no war on drugs. You know you make it uncomfortable for those people to sell drugs and they kill each other over it or stop doing it. Go find something else*	*I’m not saying its not illegal to punch the nazi I’m just saying its not wrong*
Fairness	*Because of what he did, he is up in Virginia serving time for it*	*I wish to live in a world where freedom, liberty and justice for all is a real thing*
Cheating	*Yeah that’s because they steal the money, man. They always say like, we’re going to use this for that and then it’s like, where did the money go? And it’s like, I don't know. Look at the Georgia lottery and how it’s supposed to fund all the it’s like*	*Thinking a lot about the injustice of respectability politics and the importance of the dignity of risk*
Loyalty	*But, I respect your decision and I’ll leave you alone about that, alright. But I’m waiting for you. I could use my wing man, we’re like those two little musk rats in fucking Ice Age. What? Yeah? Fucking A can’t be my wing man he is never home now*	*This resistance is not a rebellion against the nation or an act of treason but one of the deepest patriotism*
Betrayal	*That’s crazy. Can you imagine you get a call from your husband or wife "oh um you know how I went to that lame TV show that you hate yeah well I just won a trip to Paris, and I’m not taking you."*	*He is anti american anti israel and evil we should throw him out like the british did*
Authority	*You’re already in trouble and you’re getting punished tonight, you’re going to have to go to the manager*	*The appearance of the law must be upheld especially while it’s being broken*
Subversion	*New York they do. Right. Right. It’s illegal, that’s right*	*This government has shown itself entirely incapable of managing its ministers personal greed*
Purity	*I’m doing what Jesus the Lord told me to do. I’m going to be alright. And what that would have you. And just like you said, can we separate that? We … prepare for the afterlife on Earth while we live in this crazy, evil world. And we can, we have faith, like ok it’s going to be alright, you know. It’s uh temporary*	*Happy to give god bless the survivors do not be overcome by evil but overcome evil with good*
Degradation	*Ew. That looks disgusting. Just take a look. Ew*	*Dogs weren’t created to fight each other for people’s entertainment this is sickening*

#### Annotation of the EAR dataset

Each document was annotated by at least three undergraduate research assistants who were trained with the Moral Values Coding Guide^24^ with the addition of “thin morality” (e.g., “wrong”, “right”, “good”, “bad”) as a new category^2^. This moral values coding guide, used in previous studies to guide annotation of moral rhetoric in social media posts, contains instructions and numerous examples detailing how posts are differentiated as either “moral” or “non-moral,” and furthermore how moral posts are differentiated between 10 categories of moral sentiment. The ten categories are derived from the typology in the MFT (see above), which contains “vice” and “virtue” dimensions for each of the five moral foundations. In this study, in order to have a better estimate of moral language in everyday life, we did not rely on machine-learning models and hand-annotated the entirety of the transcribed language data. We first took inter-rater agreement on the “moral” label (vs. “non-moral”), then we proceeded to finer-grained moral content labels. For this reason, the “moral” posts were more frequent than the sum of all labels because annotators could agree that a post is moral, but disagree on the moral content (e.g., “care” vs. “fairness”).

#### Inter-rater agreement

Annotators’ agreement with each other in assigning moral labels to posts is shown in Table 2. Prevalence-adjusted bias-adjusted Kappas (PABAKs; Byrt et al.^35^) were computed, which adjust for imbalanced datasets (i.e., the vast majority of posts in this dataset are non-moral) by decreasing the weight of “expected” agreement. Overall, the total PABAK reliability index was 0.80 in the aggregate sample which is high compared with prior work in moral psychology^24^. Evidently, the care and cheating labels were more agreed-upon than others, suggesting that these two types of moral language are more unambiguous and easy-to-detect by human coders.

**Table 2:** Inter-rater agreement for annotations of the EAR dataset (Study 2).

	Sample 2A	Sample 2B	Sample 2C	Sample 2D	All Samples
Instances	17,307	9824	16,874	6953	50,961
Annotators	4	4	4	3	10

Label	Prevalence-adjusted bias-adjusted Kappas				
Moral	0.73	0.75	0.85	0.91	0.80
Care	0.92	0.96	0.96	0.97	0.95
Harm	0.96	0.98	0.97	0.99	0.97
Fairness	0.98	0.96	0.99	1.00	0.98
Cheating	0.97	0.95	0.99	0.99	0.98
Loyalty	0.99	1.00	0.97	0.99	0.99
Betrayal	0.99	0.99	0.99	1.00	0.99
Authority	0.95	0.98	0.98	0.98	0.97
Subversion	1.00	1.00	1.00	1.00	1.00
Purity	0.97	0.98	0.99	0.99	0.98
Degradation	0.90	0.92	0.98	0.99	0.94

### Results

#### Distribution of moral posts

**Figure 2:**
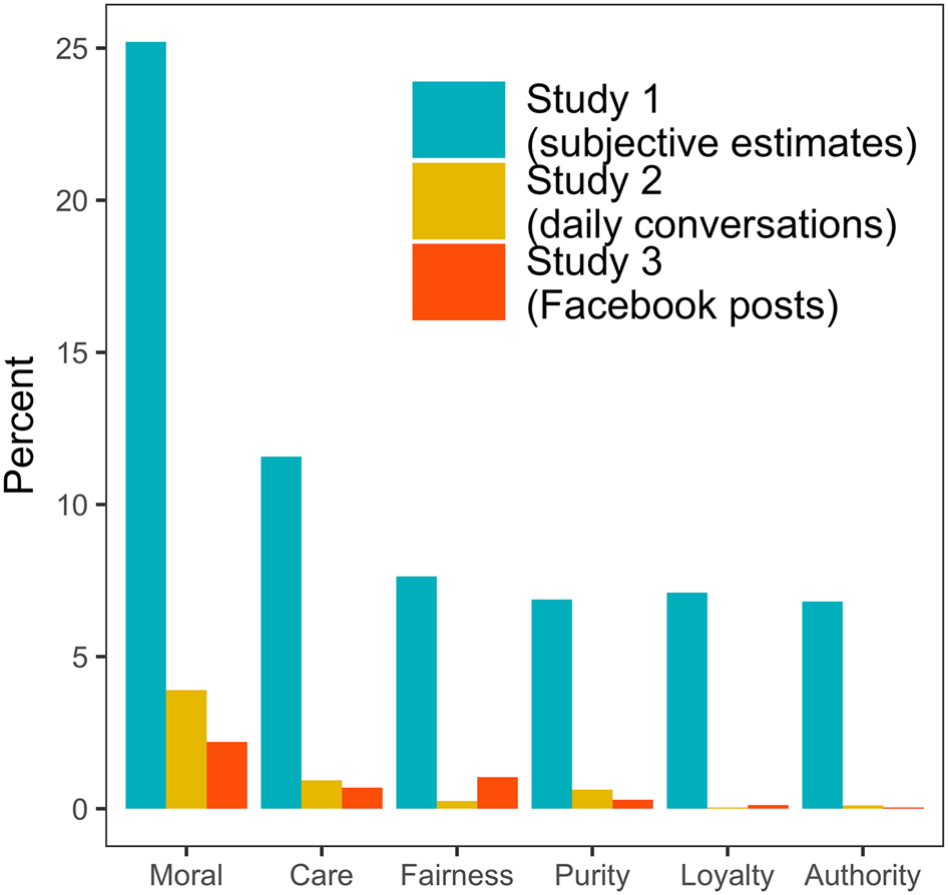
The estimate of moral concerns in all studies.

The total distribution of moral snippets per category in each sample are shown in Supplementary Materials. Across all 50,961 snippets of audible linguistic behavior, the frequency of moral content, coded according to MFT, was surprisingly low, 3.9%, and considerably lower than self-reports pertaining to the same MFT categories in Study 1 (see Fig. 2). Adding content coded as “thin morality” did not substantially change this picture (0.8%). Thin morality is another type of moral language which is more general and abstract than the moral foundations, and manifested in using terms such as “right,” “bad,” “wrong,” and “ought”^2^.

#### Language content in moral snippets

**Figure 3:**
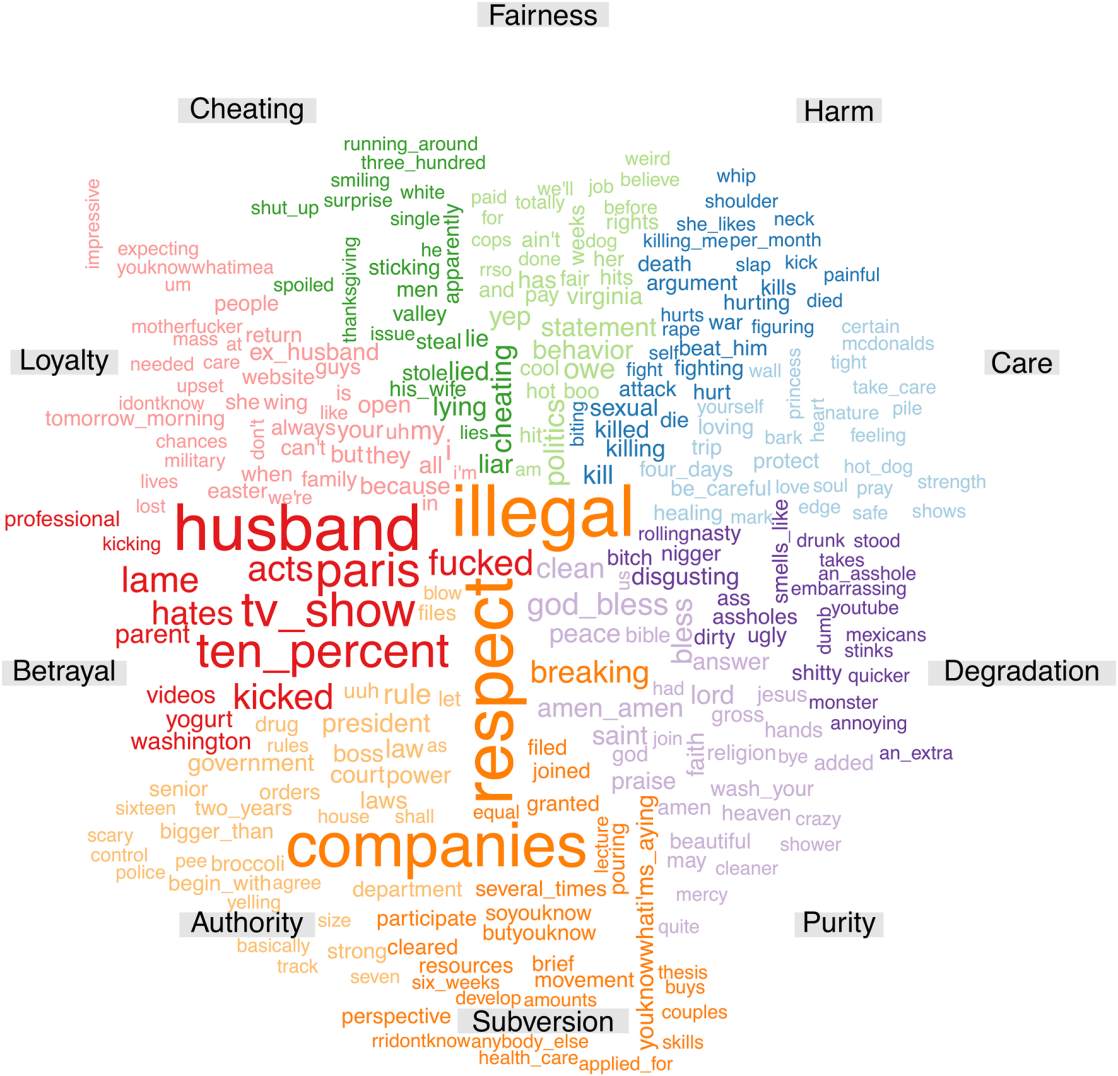
Visualization of the most salient words used in each category within moral posts in the EAR dataset (Study 2). Colors indicate the respective category as marked by each label, while the size of words is proportional to their weight in predicting the label across posts.

Here we visualize the most salient words for morally labeled instances in the EAR dataset. Instances were represented as term frequency-inverse document frequency (TF–IDF) vectors^36^ and feature importance scores were extracted from a cross-validated Support Vector Machine (SVM) classifier^37^, for each category. The most highly weighted features per category are shown in Fig. 3 (since women and men differ in moral concepts such as the self-importance of moral identity^38^, we present women’s and men’s weighted features per category in Supplementary Materials).

Overall, only 3.9% of everyday talk was identified as having moral aspects (the effects of age and gender^39^ are presented in Supplementary Materials). With regard to the composition of moral talk, care dominated, and in contrast to the bulk of earlier findings^26,40^, the frequency of purity exceeded the frequency of fairness. This study’s descriptive results indicate that morality might be substantially less frequent in everyday conversations than presumed when people are explicitly asked to estimate its prominence.

## Study 3

Complementing our analysis of face-to-face interactions, we assessed the frequency and composition of moral talk in private Facebook posts using a machine learning algorithm trained on expert annotations of moral foundations in 6991 Facebook posts. This study was designed to further examine how prominent moral language is in people’s communications on social media.

### Methods

#### Participants and facebook data

Participants were recruited via the *yourmorals.org* platform, through which users provided access to their private Facebook posts for research purposes. Initially, 4414 participants’ data were recorded, and Facebook posts were retrieved via the Facebook application programming interface (API) with the approval of Facebook and the approval of the IRB at the University of Southern California. We used preprocessing criteria consistent with prior work^27^, leaving 3822 participants (*Min*_*age*_ = 18; *Max*_*age*_ = 65). After preprocessing posts via the Natural Language Toolkit (NLTK) in Python (3.6.x), during which hyperlinks and non-word tokens were removed, short posts (fewer than 5 tokens) and non-English posts—as predicted by the *langdetect* (1.0.x) Python library—were also removed. This procedure reduced the total number of posts from 165,787 to 111,886, and the number of participants to 3643. In terms of the contents of participants’ Facebook posts, posts overall contained an average of 28.6 tokens (*SD* = 34.4). On average, participants had 30.7 posts (*SD* = 24.2). From this larger dataset, a smaller set of posts was randomly sampled in order to perform annotation of moral labels.

Based on findings of Study 2, it was expected that moral labels would be relatively rare in this dataset, hence a true random sample of posts (*n* = 3500) and a “stratified” random sample of posts (*n* = 3500) were combined, where stratification was performed based on the approximate moral content of each post according to their semantic similarity with the explicit moral lexicon (see below).

#### Annotation of facebook posts

To estimate the moral content of each post in the “stratified” sample, we used the Moral Foundations Dictionary (MFD)^41^, with vice and virtue categories collapsed into 5 categories corresponding to the five foundations (i.e., care, fairness, loyalty, authority, and purity). Next, distributed dictionary representations (DDR)^42^ were calculated for each post and each foundation. DDR uses a pre-trained latent semantic representation of the vocabulary (i.e., word embeddings, in this case Global Vectors for Word Representation [GloVe])^43^. Word embeddings, which are learned from large text corpora, contain fine-grained semantic information about the meaning of words, in particular the relationship among similar words. Here, GloVe was used to compare dictionary representations (element-wise averages of word vectors for each word in a dictionary) to document representations (averaging word vectors of the document) via cosine similarity. As a result, each document is represented by its DDR vector, in which each feature demonstrates a dictionary’s loading on (i.e., similarity to) the document. DDR vectors have been shown to be more effective than word counting in predicting actual moral content using the MFD^42^. For each foundation, 700 posts were randomly sampled (without replacement across foundations) from the 5 percent of documents with highest dictionary loading values, with the expectation that higher loadings are more likely to contain moral content. These 7000 posts were then hand-annotated for moral content. See Table 1 for examples.

Annotators were research assistants trained via an existing coding manual for identifying fine-grained expressions of moral concern in natural language^24^. Each post was annotated by a minimum of 3 annotators for being “moral” vs. “non-moral” and in case the post was “moral,” the annotators chose among more nuanced moral labels (*n* = 10) that were not mutually exclusive. The annotators had the option to annotate probabilistically, that is, when they were not absolutely sure but inclined toward choosing a label, they could use a “maybe” option. To convert the set of annotations for each text into binary labels that can be utilized in a language classification model, the majority vote of each label was taken. We first took inter-rater agreement on the “moral” label (vs. “non-moral”), then we proceeded to more nuanced moral content labels. For example, if two annotators labeled a post as “care” and another did not, this post would be taken to contain care language (i.e., a “positive” label for “care”).

#### Automated label classification

To train a machine-learning algorithm which automatically generates moral labels for the entire corpus of Facebook posts (*N* ~ 111,000) given a smaller annotated sample (final *n* = 6983 posts), we applied language model fine-tuning^44^. Language model fine-tuning refers to the process of adapting a previously trained language model, which itself is a probabilistic model of language (e.g., the conditional probability of one word occurring given its surroundings), to a specific language understanding task, such as text classification. In this case, we fine-tune (i.e., adapt) the state-of-the-art pretrained language model, the Bidirectional Encoder Representations from Transformers (BERT)^45^ to the classification of moral labels. We used the *transformers* (version 3.1.x) library^46^. This model has 12-layers, 768 hidden units, 12-heads, 110 M parameters and was trained on lower-cased English text. To fine-tune the BERT architecture to predict the ten target labels, we followed standard procedure in the natural language processing (NLP) literature. Specifically, the goal was to train a multi-label prediction layer *h*_*labels*_, appended to the output of the pre-trained BERT model, *g* (*x*), which is the hidden representation for the input, *x*. During fine-tuning, the weights (parameters) of both the pretrained 12-layer BERT model, *g*, and the classification layer, *h*, are updated based on the accuracy of the predicted labels, {*f*_*care*_ (*x*), *f*_*harm*_ (*x*), *f*_*fairness*_ (*x*), …, *f*_*purity*_ (*x*)}, during batch-training with the annotated dataset.

Posts were preprocessed and tokenized using the preexisting BERT tokenizer in the *tokenizers* library (Wolf et al.^46^), which splits sentences into a collection of token representations understandable by the BERT model. Using 10 binary labels per post representing the expressions of moral foundation, collected during annotations, we conducted multi-label classification: in other words, the fine-tuned model generated probabilities of each of the 10 labels occurring in each input text.

The extreme sparsity of the moral labels in our dataset makes the task of prediction more challenging, motivating additional training steps. Before fine-tuning on the data, we performed an initial fine-tuning process using the Moral Foundations Twitter Corpus (MFTC)^24^, and we further fine-tune the model on our Facebook dataset. The MFTC contains approximately 35,000 Twitter posts with annotated moral labels based on the typology of moral language by MFT, and was used as an auxiliary dataset for training our model in the present work. After selecting the majority labels at the post-level, we evaluate the fine-tuning process in a tenfold cross-validation. We select the fine-tuned model on MFTC with the highest macro F_1_ score on the validation set and performed the second round of fine-tuning, this time on the annotated Facebook dataset (*n* = 6983) with tenfold cross validation. All the models are fine-tuned for 5 epochs using the “Adam” optimizer^47^ on an NVIDIA GeForce RTX 2080 SUPER. Each epoch of fine-tuning on MFTC and Facebook took approximately 4 min and 1 min, respectively.

Finally, to predict the presence of each label on the remaining Facebook posts (*n* ~ 111,000)**,** predictions were generated, for each of the 10 Facebook models (i.e., one per training fold from cross validation). The result of these predictions, consisting of 10 predicted labels per moral label, was then aggregated via majority vote. More specifically, we predict the presence of each foundation only in cases that at least half of the models agree on that specific label.

#### Inter-rater agreement

All PABAKs are shown in Table 3. Traditionally, acceptable ranges of PABAK are similar to acceptable ranges of other inter-rater reliability indices, with values over 0.6 indicating adequate reliability. Here, the label imbalance of our dataset borders on the extreme (i.e., more than 95% of documents labeled as non-moral), thus inter-rater agreement coefficients are correspondingly more difficult to interpret. Still, some caution should be exercised in interpreting these results as an overall PABAK of 0.59 (for moral vs. non-moral labels) is not quite high.

**Table 3:** Inter-rater agreement for 6983 facebook posts (Study 3).

Label	*PABAK*
Moral	0.59
Care	0.91
Harm	0.94
Fairness	0.89
Cheating	0.87
Loyalty	0.93
Betrayal	0.95
Authority	0.98
Subversion	0.95
Purity	0.95
Degradation	0.93

#### Classification performance

The performance of machine-learning models across the 10 cross validation folds for fine-tuning models on the MFTC and Facebook datasets were acceptable (see Supplementary Materials). A single model from the MFTC fine-tuning stage was selected based on the highest F_1_ score, averaged across labels. The F_1_, precision, and recall metrics from tenfold cross-validation are available in Supplementary Materials.

### Results

#### Distribution of moral posts

Our models automatically labeled 111,886 Facebook status updates in this study. Results indicated that only 2.2% of the posts contained moral language, overall. Turning to the composition of this moral talk, the most common moral concern was fairness/cheating (47.5% of all moral content), followed by care/harm (31.4% of all moral content). References to purity/degradation, loyalty/betrayal, and authority/subversion were substantially lower (see Fig. 2). Age and gender differences in moral talk are presented in Supplementary Materials.

#### Language content in moral posts

**Figure 4:**
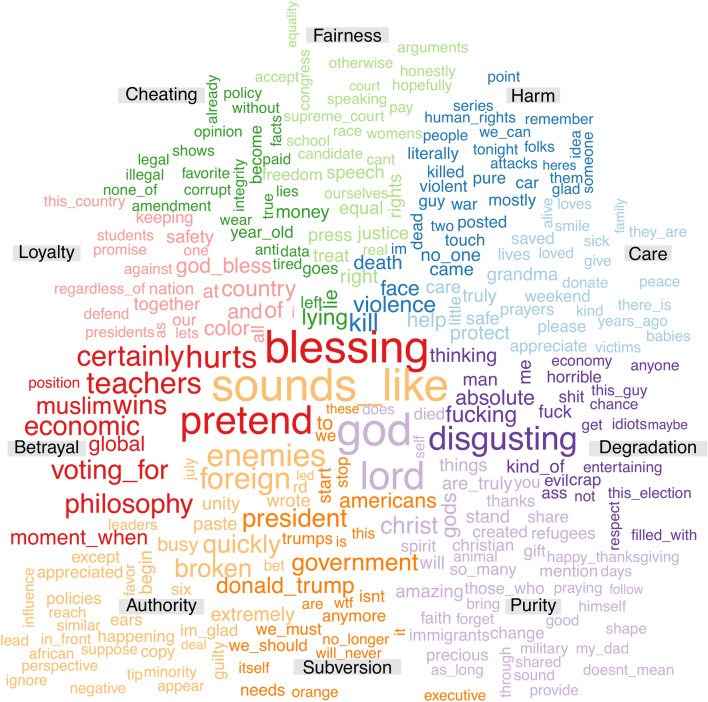
Visualization of the most salient words used in each category within moral posts (Study 3). Colors indicate the respective category as marked by each label, while the size of words is proportional to their weight in predicting the label across posts.

To better understand the content of each moral category, word clouds were generated using predictive word-level features. Specifically, TF–IDF was used to quantify each post as a vector of normalized word counts, and SVMs with linear kernels were trained to predict each of the 10 labels, respectively. Models were trained similarly to Study 2. The terms based on extracting the most highly predictive word features per category are shown in Fig. 4.

## General discussion

We reported three studies designed to help empirically evaluate the widely held assumption that morality occupies a position of singular prominence in people’s everyday talk. This assumption is supported by religious and philosophical tradition, and compatible with findings in moral psychology that suggest that moral considerations occupy a privileged position in the assessment of both self and others^15^. Yet it remains an open question whether this perceived importance is associated, as might be expected, with an elevated prominence in a key building block of daily life, people’s everyday discourse: do moral considerations make frequent appearance in people’s daily interactions?

Paralleling prior work^18^, our participants’ self-reports (Study 1) suggest that between 20 and 30% of everyday talk is thought to have moral content, apparently confirming the prominence-of-morality hypothesis. In stark contrast, moral talk turned out to be surprisingly rare in large samples of random snippets of everyday conversations (4.7%; Study 2) and private Facebook postings (2.2%; Study 3). Despite these discrepancies between participants’ subjective frequency estimates and the objectively observed frequency estimates, the *content* of moral talk is comparable across methods. In our studies, like the Hofmann et al. study^18^, care and fairness concerns dominated, with the exception of an elevated frequency for purity in Study 2, which was largely driven by the subsample in which participants wore the EAR before and after a meditation intervention, possibly increasing spiritual talk. This is consistent with prior results that documented a high endorsement of the “individualizing” values of care and fairness (e.g., compassion and social justice) in Western societies^48^.

Cognitive, motivational, and social factors are likely to contribute to the observed discrepancies between the self-reported and observed frequency of moral talk. Frequency questions elicit a confirmatory search for relevant instances that results in overestimates when a few salient examples easily come to mind^49^. Related questionnaire material, from the introduction to preceding questions, can increase the accessibility of topical information^50,51^. Moreover, as the “availability heuristic” indicates^52^, people estimate the frequency of an event, or the likelihood of its occurrence, by the ease with which instances come to mind. Accessible instances may include what one imagined saying, or wanted to say, but didn’t, which are often conflated^53^. Detailed questions about moral concerns may also invite participants to present themselves in the most favorable moral light, inducing a self-enhancing bias^54^. Observational assessments of the moral content of natural language avoid or attenuate these biases, resulting in lower frequency estimates for moral talk.

While these estimates are probably closer to the actual frequency of moral *talk,* not all private moral thoughts and feelings are expressed in public moral talk, as observed in Studies 2 and 3. Our observations of public moral talk, on the other hand, do not allow us to assess participants’ “inner” experience. But neither the self-report nor the observational measures need be thought to deliver misleading estimates; rather, they may be measuring different aspects of moral life, neither of which should necessarily be considered more important than the other. Nevertheless, the paucity of morality in observable everyday talk stands in stark contrast to lay intuitions and self-reports, as well as scholars’ emphasis, on the singular relevance of morality in everyday life.

This observation both underscores an important limitation of the present research and suggests directions for future work. Linguistic behavior, while undoubtedly important, is only one aspect of moral functioning, and its relationship to other aspects of moral functioning is uncertain. We have already intimated that words are not thoughts, and neither are words deeds: what people are saying does not tell us all that they are thinking, or all that they might be expected to do. With respect to moral action, it remains true that “talk is cheap,” and linguistic behavior cannot provide definitive measures of more concrete behavior, like donating to charity, or cheating on taxes. The present work, one of the first empirical examinations of morality in everyday social interactions using naturalistic observations, is offered as an impetus to future research programs; its central finding—the frequency of moral content in everyday discourse is surprisingly low—suggests we must take seriously the possibility that morality is less relevant in people’s everyday life (or at least their daily talk) than is commonly supposed.

If it in fact obtains, this circumstance would have both theoretical and practical implications of considerable import. Theoretically, it would suggest that time-honored philosophical and religious doctrines upholding the singular, overriding, importance of morality are inconsistent with the importance people actually impugn to morality in everyday life. This does not, of course, show that such theories are wrong—many powerful theories run afoul of common sense—but it does indicate that such theories have a hitherto unappreciated explanatory burden: accounting for why people appear not to assign the relevance to morality that philosophical and religious traditions require. Further research, using methodologies other than the linguistic ones employed here, such as experimental work on the weight assigned moral considerations in judgment and decision making, is required, if we are to better understand the role morality plays in everyday life.

The paucity of morality in Facebook status updates is interesting considering the fact that moral language has been found to be particularly powerful in motivating people to take action. Social media posts that contain moral words are also more likely to “go viral” and receive attention from users^17,55^. However, moral rhetoric might be exaggerated in platforms like Twitter, which typically differ from daily conversations through a higher intention of reaching a broad audience. In social-media platforms like Twitter, politicians and organizers of social movements often express moral concerns in an effort to increase online engagement and to influence perceived norms within social networks. In such contexts, using moral language in a post has been found to stimulate moral engagement and sharing, whereas using “too much” moral language reduces engagement and sharing, a phenomenon termed “moral penalty”^56^. In the present research, we were primarily interested in the frequency of moral language in daily communication, not in the use of moral rhetoric in public persuasion. Hence, we relied on private Facebook updates rather than tweets. Future research may fruitfully examine the frequency and nature of moral language across different social media platforms.

Finally, we mention three constraints on generality of the present descriptive findings for replication and follow-up studies^57^. First, our three studies include only U.S. participants and our samples were not representative. Therefore, our estimates cannot be generalized to other cultures, especially less WEIRD (Western, Educated, Industrialized, Rich, and Democratic^58,59^) populations wherein moral norms tend to be tighter^60^ (i.e., transgressions of moral norms are strictly punished), intentions are less important in moral judgments^61,62^, and group-oriented moral values such as conformity and obedience are more strongly endorsed^39,48,63^. For instance, people in tighter and less-WEIRD cultures may be less likely to talk about their non-normative values, but more likely to frequently talk about their norm-affirming values to signal conformity and avoid ostracization (i.e., if some moral behaviors are expected, only violations would be talked about, along with unexpected acts of unusually costly moral behavior). Morality may guide day-to-day actions (and reasoning) by constraining what seems possible to do in a particular environment^64^. Moreover, our linguistic analyses focused on English, which has been proposed to be a peculiar language compared with the substantial linguistic diversity observed around the globe^65^. Language can have downstream effects on seemingly non-linguistic judgments and behaviors^65^; hence, it is desirable for future research to replicate these results in less-WEIRD cultures and in non-English-speaking populations. Second, we note that the original five-dimensional typology of moral values by MFT may not cover the entire moral domain. For example, researchers have proposed Liberty^66^ and Honor^67^ as potential candidates to be included in MFT. Recently, MFT theorists proposed that Fairness can be split into two distinguishable foundations of Equality and Proportionality^68^. Future research is encouraged to include these additional foundations as constituents of moral language in examining the paucity of moral talk in everyday life. Third, in our EAR samples, we did not collect information about who people were with when recordings took place. This is a limitation since prior work has established that context influences the importance of moral values^69^.

## Supplementary Information

Supplementary Information.

## Data Availability

The data (except for identifiable recordings and private Facebook posts), code, and materials reported in the manuscript are publicly available. All materials can be found at https://osf.io/gcvyu/. Mohammad Atari should be contacted for data requests.
