# Supplementary material for: The paucity of morality in everyday talk
Source: Sci Rep. 2023 Apr 12;13:5967. doi: 10.1038/s41598-023-32711-4 (PMC10097712; doi:10.1038/s41598-023-32711-4)
Supplement: Supplementary file 1 — Supplementary Information. [file 41598_2023_32711_MOESM1_ESM.docx]

**Supplementary Materials**

**Study 1**

**Results**

Density plots (with mean, median, and mode) for these two questions are presented in Figure S1. With regard to benchmarking morality against other everyday topics using the slider response option (Sample 1A), descriptive statistics are presented in Table S1. Benchmarking statistics using the pie chart response option (Sample 1B) are presented in Table S2.

**Figure S1**

*Density Plots for Perceived Prevalence of Morality in Everyday Conversations*

**
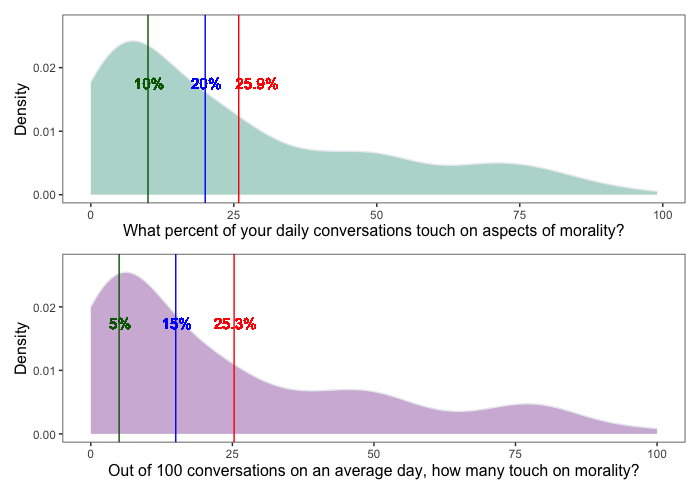
**

**Table S1**

*Descriptive Statistics for Benchmarks (Study 1, Sample 1A)*

**
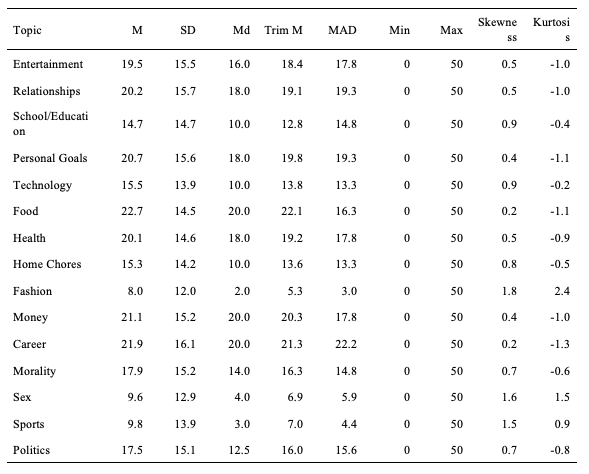
**

**Table S2**

*Descriptive Statistics for Benchmarks (Study 1, Sample 1B)*

**
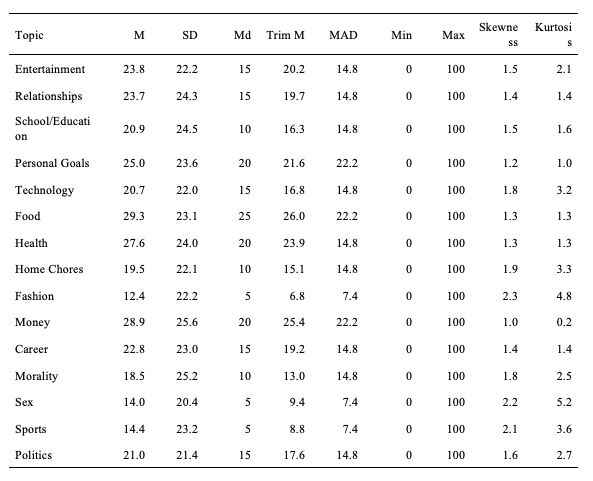
**

**Inferential Statistics.** We tested the hypothesis of equal trimmed means using a robust percentile bootstrap method (Mair & Wilcox, 2020) in Samples A and B. We used 5,000 bootstraps to calculate trimmed means (γ = 0.2) between 15 topics. The trimmed means were significantly different across groups based on an effective number of 5,000 bootstraps (*F* = 66.81, *p* < .001). Here, we only report differences between the perceived prevalence of morality in conversations with other topics. In Sample A, morality was found to be perceived as conversationally more salient than fashion (95%CI = [7.98, 14.72], *p* < .001), sex (95%CI = [6.23, 13.38], *p* < .001), sports (95%CI = [6.56, 14.13], *p* < .001). In addition, morality was perceived to be less frequent than food (95%CI = [2.35, 11.07], *p* < .001), career (95%CI = [1.21, 11.21], *p* = .002). The other pairwise comparisons were not statistically significant (*p*s > .099). In Sample B, trimmed means were significantly different across 15 topics (*F* = 24.04, *p* < .001). Particularly, morality was perceived to be more conversationally prevalent than fashion (95%CI = [1.24, 10.73], *p* = .002). On the other hand, morality was perceived to be less conversationally frequent than food (95%CI = [7.53, 20.85], *p* < .001), personal goals (95%CI = [3.43, 16.06], *p* < .001), money (95%CI = [5.60, 20.31], *p* < .001), career (95%CI = [0.39, 13.00], *p* = .027), entertainment (95%CI = [2.15, 14.20], *p* = .001), health (95%CI = [5.39, 17.89], *p* < .001), politics (95%CI = [0.06, 10.61], *p* = .045), and relationships (95%CI = [0.56, 13.09], p = .020). The other pairwise comparisons were not statistically significant (*p*s > .310).

**Study 2**

**Results**

The distribution of moral values in Study 2 are presented in Figure S2. As can be seen in the descriptive statistics, moderate variation exists between samples 2A through 2D. The most highly weighted features per category for women and men are shown in Figures S3 and S4, respectively. Finally, the effects of age and gender on moral talk, vices and virtues, are respectively presented in Figure S5 and S6.

**Figure S2**

*Distributions of Each Moral Label (Majority Vote) in the EAR Dataset*


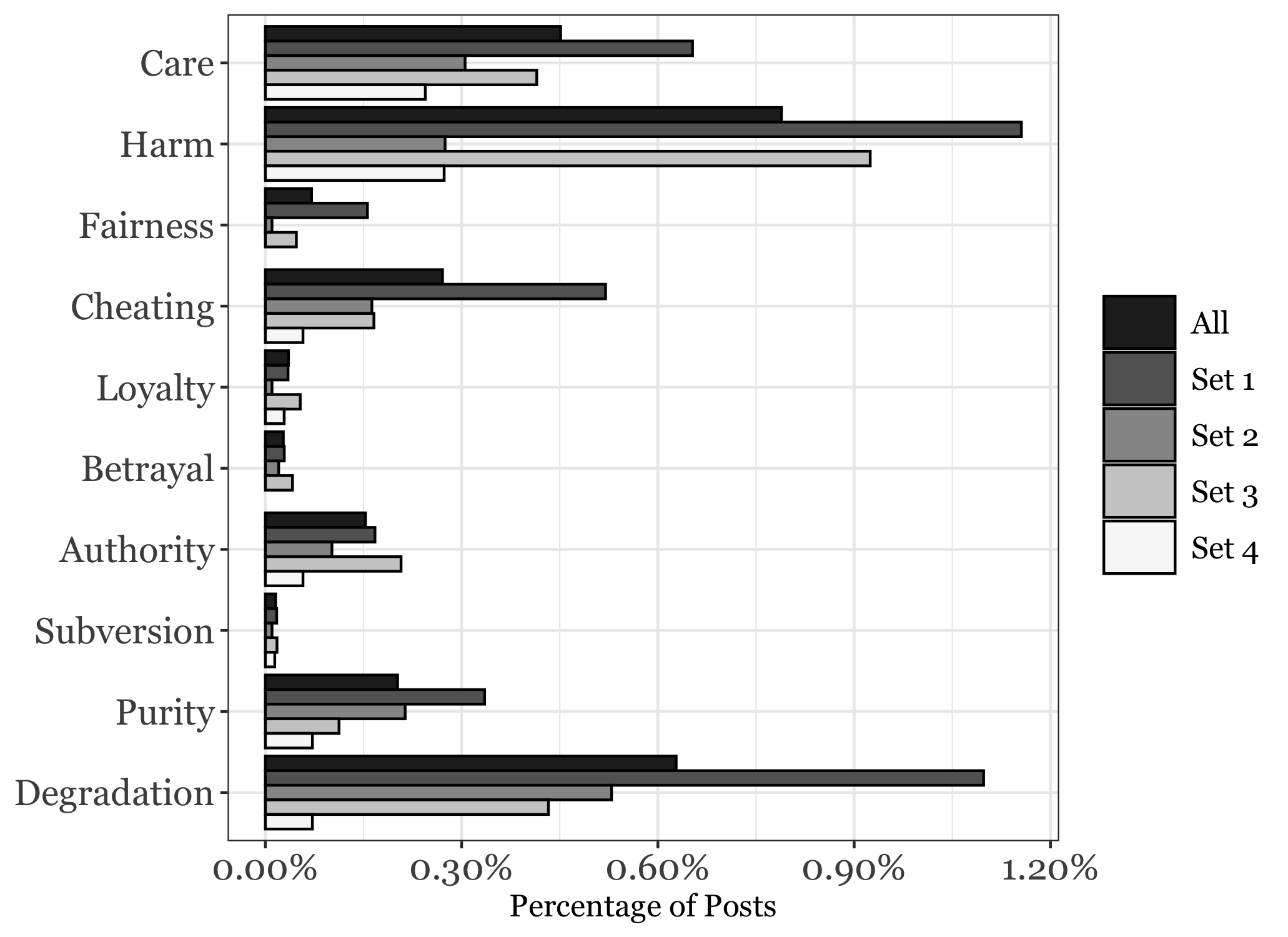


Figure S3

*Visualization of the Most Salient Words for Women used in Each Category (Study 2)*


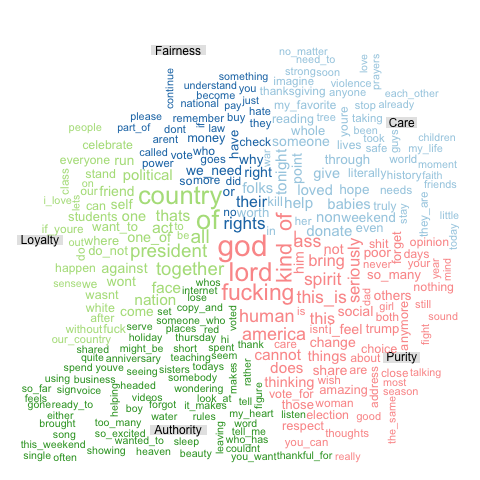


Figure S4

*Visualization of the Most Salient Words for Men used in Each Category (Study 2)*


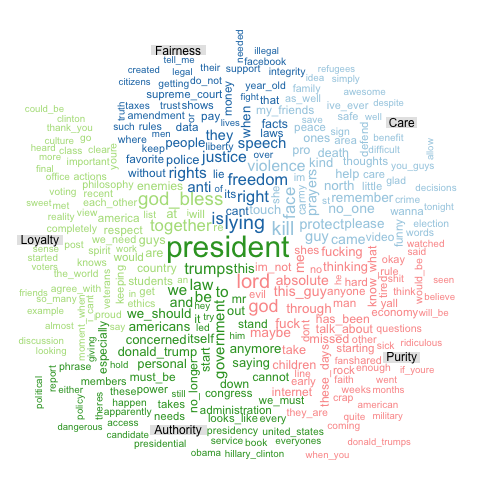


Figure S5

*Incidence Rate Ratios for Poisson Models Predicting the Number of Posts (Vices) Based on Age and Gender. Bars are 95% Confidence Intervals*


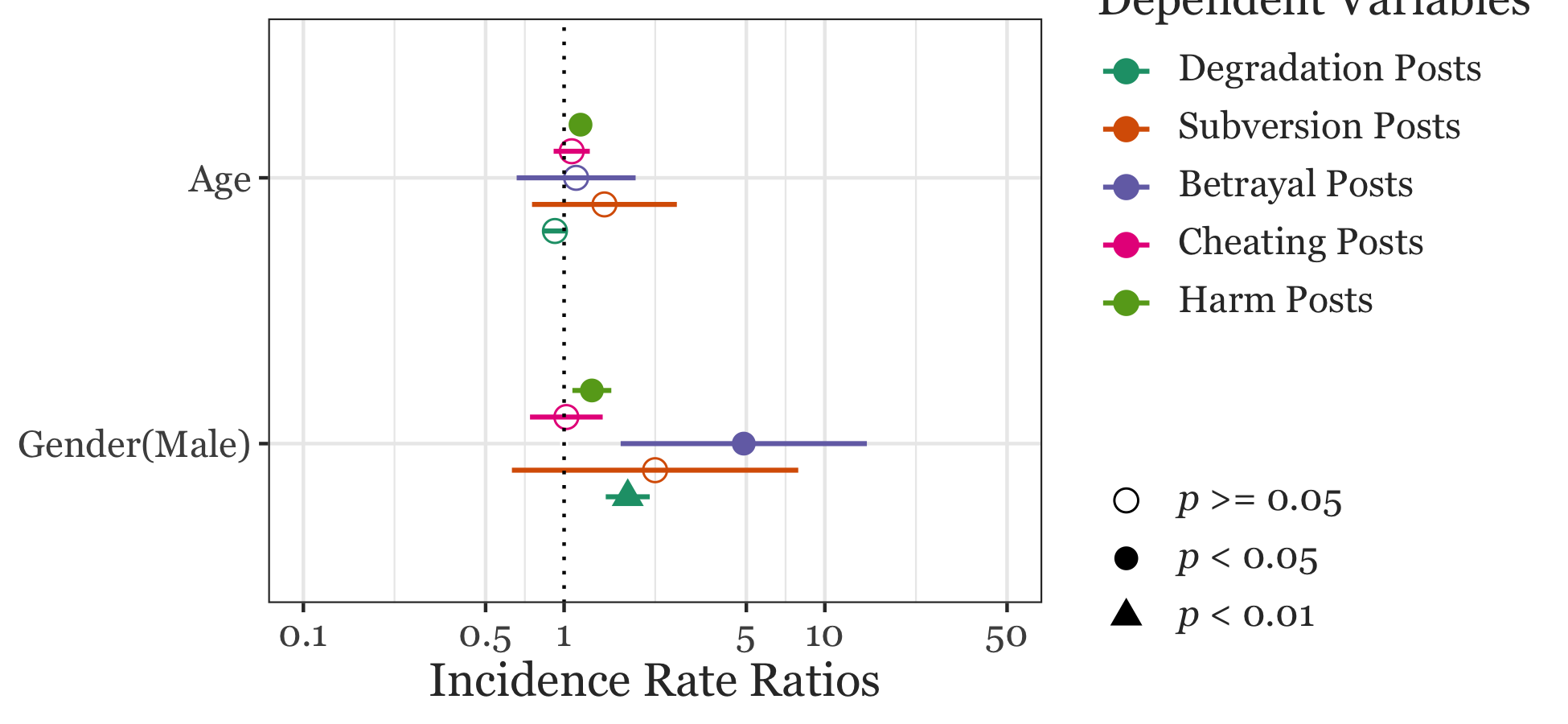


Figure S6

*Incidence Rate Ratios for Poisson Models Predicting the Number of Posts (Virtues) Based on Age and Gender. Bars are 95% Confidence Intervals*


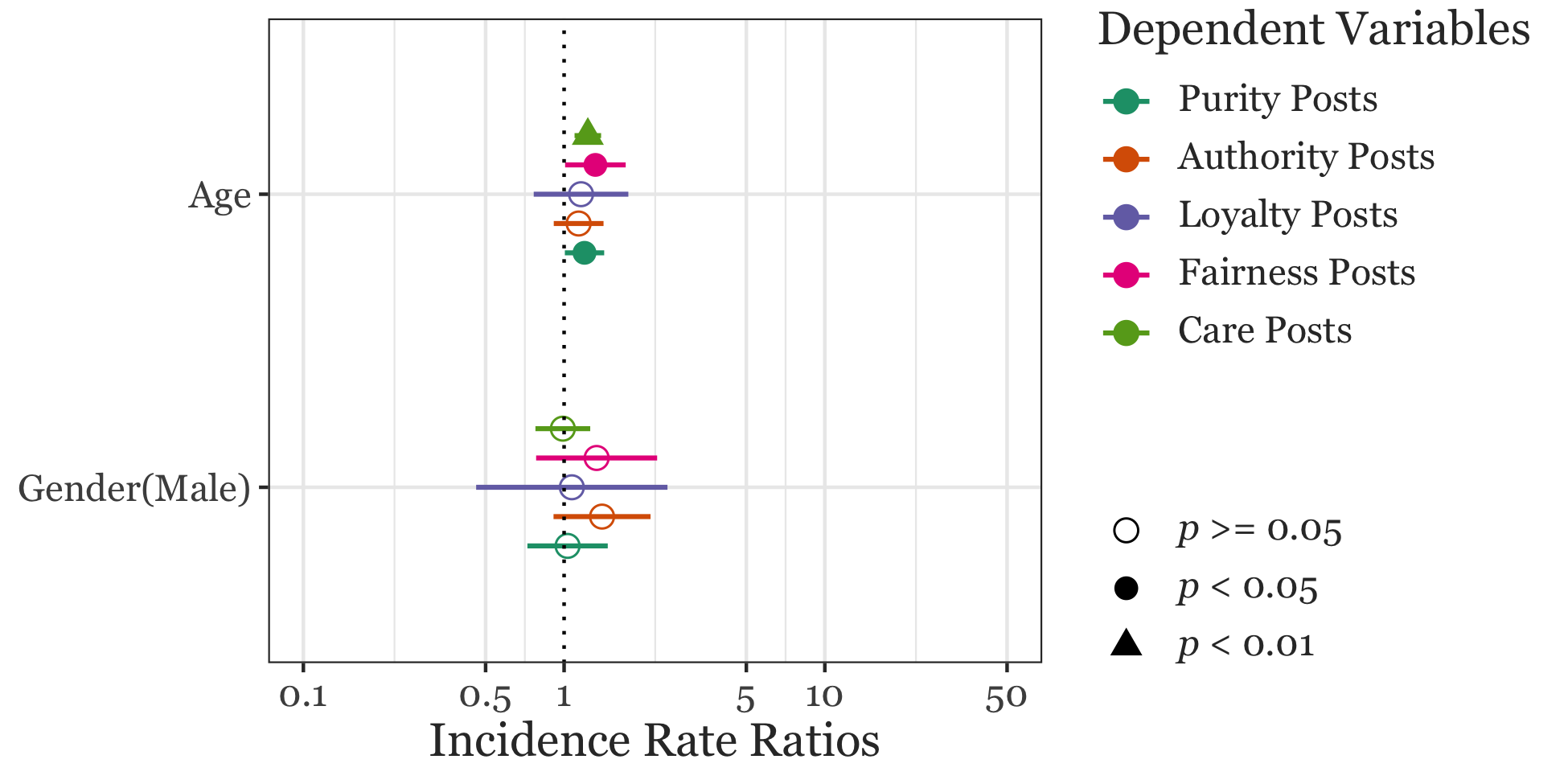


**Study 3**

**Methods**

The average performance of machine-learning models across the 10 cross validation folds for fine-tuning models on the MFTC and Facebook datasets are shown in Table S3.

**Table S3**

*Classification Performance for Fine-Tuned Multi-Label Models (Study 3)*

|  | MFTC | | | Facebook | | |
| --- | --- | --- | --- | --- | --- | --- |
|  | Precision | Recall | F_1_ | Precision | Recall | F_1_ |
| Care | 0.71 (0.05) | 0.72 (0.07) | 0.71 (0.02) | 0.43 (0.18) | 0.41 (0.24) | 0.40 (0.18) |
| Harm | 0.68 (0.04) | 0.60 (0.04) | 0.64 (0.02) | 0.31 (0.24) | 0.27 (0.21 | 0.28 (0.22) |
| Fairness | 0.78 (0.05) | 0.75 (0.03) | 0.77 (0.02) | 0.49 (0.27) | 0.32 (0.28) | 0.36 (0.27) |
| Cheating | 0.69 (0.05) | 0.63 (0.06) | 0.65 (0.03) | 0.61 (0.18) | 0.37 (0.16) | 0.45 (0.17) |
| Purity | 0.60 (0.10) | 0.48 (0.11) | 0.52 (0.08) | 0.32 (0.41) | 0.26 (0.30) | 0.26 (0.30) |
| Degradation | 0.72 (0.07) | 0.55 (0.07) | 0.62 (0.05) | 0.39 (0.44) | 0.19 (0.18) | 0.24 (0.24) |
| Loyalty | 0.77 (0.05) | 0.65 (0.03) | 0.71 (0.03) | 0.34 (0.39) | 0.29 (0.33) | 0.29 (0.31) |
| Betrayal | 0.56 (0.06) | 0.54 (0.07) | 0.55 (0.04) | 0.10 (0.32) | 0.05 (0.16) | 0.07 (0.21) |
| Authority | 0.70 (0.07) | 0.61 (0.07) | 0.65 (0.04) | 0.00 (0.00) | 0.00 (0.00) | 0.00 (0.00) |
| Subversion | 0.58 (0.06) | 0.39 (0.06) | 0.46 (0.04) | 0.03 (0.11) | 0.05 (0.16) | 0.04 (0.13) |

**Results**

**Descriptive** **Statistics of Facebook posts**. For both subsets of Facebook posts with *annotated* labels and *machine-predicted* labels, here we detail the distribution of labels in terms of overall frequency, individual-level frequency, and covariance among moral labels. From the set of 6,983 annotated Facebook posts, 944 (13.5%) were determined by a majority of annotators to be moral. The distribution of posts labeled as each of the 10 moral categories is visualized in the left-hand side of Figure S3. We note that, since annotators disagreed on the *category* of moral labels more often than on the existence of moral content in a post, the number of “moral” posts by majority agreement is greater than the sum of each moral category.

The predicted labels for the entire Facebook dataset indicate a conservative estimate of the amount of moral language in a larger sample and is additionally used below to examine the distribution of moral posts at the individual level. These labels are considered conservative given the performance of the classifiers (see Table S3), which do not yield high recall (low false negatives). In Figure S7, the distribution of moral posts in the annotated dataset is juxtaposed with that of the predicted dataset.

**Figure S7**

*Distributions of Moral Posts for the Facebook Dataset (Study 1)*


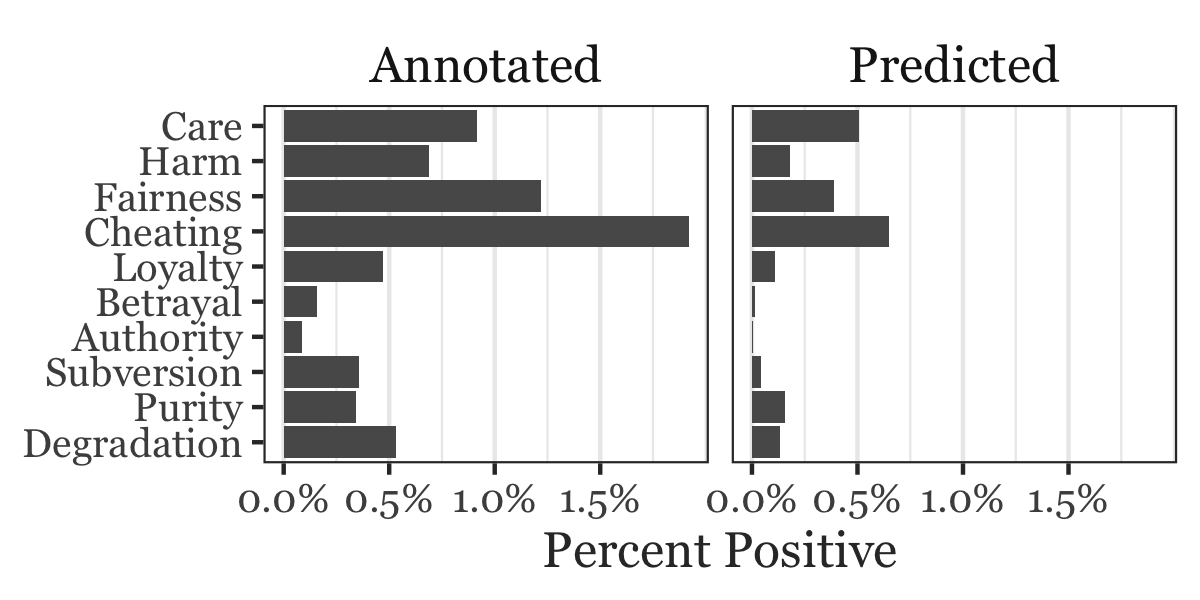


**Individual-Level Predictors of the Moral Posts**. The moral labels, predicted by the fine-tuned multi-label classifier, for the corpus of 111,886 posts, were counted for each participant (*N* = 3,643). Figure S8 shows the percentage of participants that have a given number of posts (the frequencies of participants with zero posts are not shown), for each respective label. For example, approximately 2.3% of participants have exactly 2 “care” posts.

**Figure S8**

*Frequency of Each Moral Label at Participant-Level*


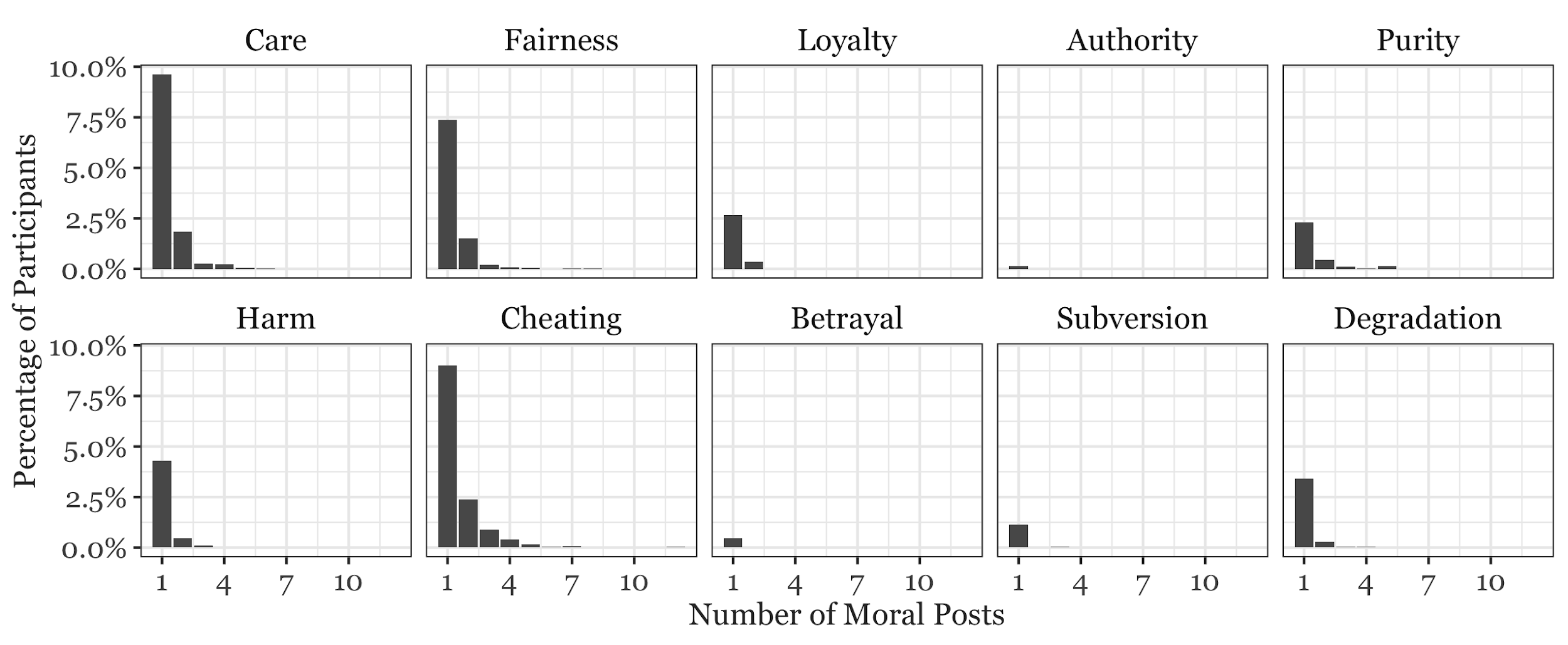


Next, participants’ responses on the Moral Foundations Questionnaire (MFQ) (Graham et al., 2011) were modeled in relation to their individual-level frequency within each moral category. Participants completed the 30-item MFQ which measures the relevance individuals ascribe to each of the foundations. Half of the items (i.e., the “Relevance” section) are rated along a 6-point Likert-type scale ranging from 0 (*Not at all relevant*) to 5 (*Extremely relevant*) and the other half (i.e., the “Judgments” section) are rated along a 6-point Likert-type scale ranging from 0 (*Strongly disagree*) to 5 (*Strongly agree*). The internal consistency coefficients for the five subscales were .70, .64, .72, .78, .87 for care, fairness, loyalty, authority, and purity, respectively.

For each of the 10 categories, a separate Poisson regression was fit with 5 predictors (MFQ scores for each foundation). Since participants had varying numbers of posts (*M* = 30.7, *Mdn* = 26.0, *SD* = 24.2), the log-transform of the number of total posts, per participant, was added as an offset to the model. Across all 10 categories, a significant relationship was found only for the care, fairness, cheating, and purity labels. The likelihood ratios for each MFQ predictor, for each of these four models, are visualized in Figure S9. Specifically, higher self-reported care concerns predicted a higher rate of care posts (β = 0.403, *SE* = 0.067, *p* < 0.001); lower self-reported authority concerns predicted higher rate of cheating posts (β = -0.168, *SE*=0.059, *p* = 0.004); and higher self-reported loyalty (β = 0.387, *SE* = 0.134, *p* = 0.004), lower authority (β = -0.389, *SE* = 0.112, *p* = 0.001), and higher purity (β = 0.955, *SE* = 0.093, *p* < 0.001) significantly predicted the number of purity posts.

**Figure S9**

*Incidence Rate Ratios for Poisson Models Predicting the Number of Posts Based on Self-Reported Moral Values. Bars are 95% Confidence Intervals*


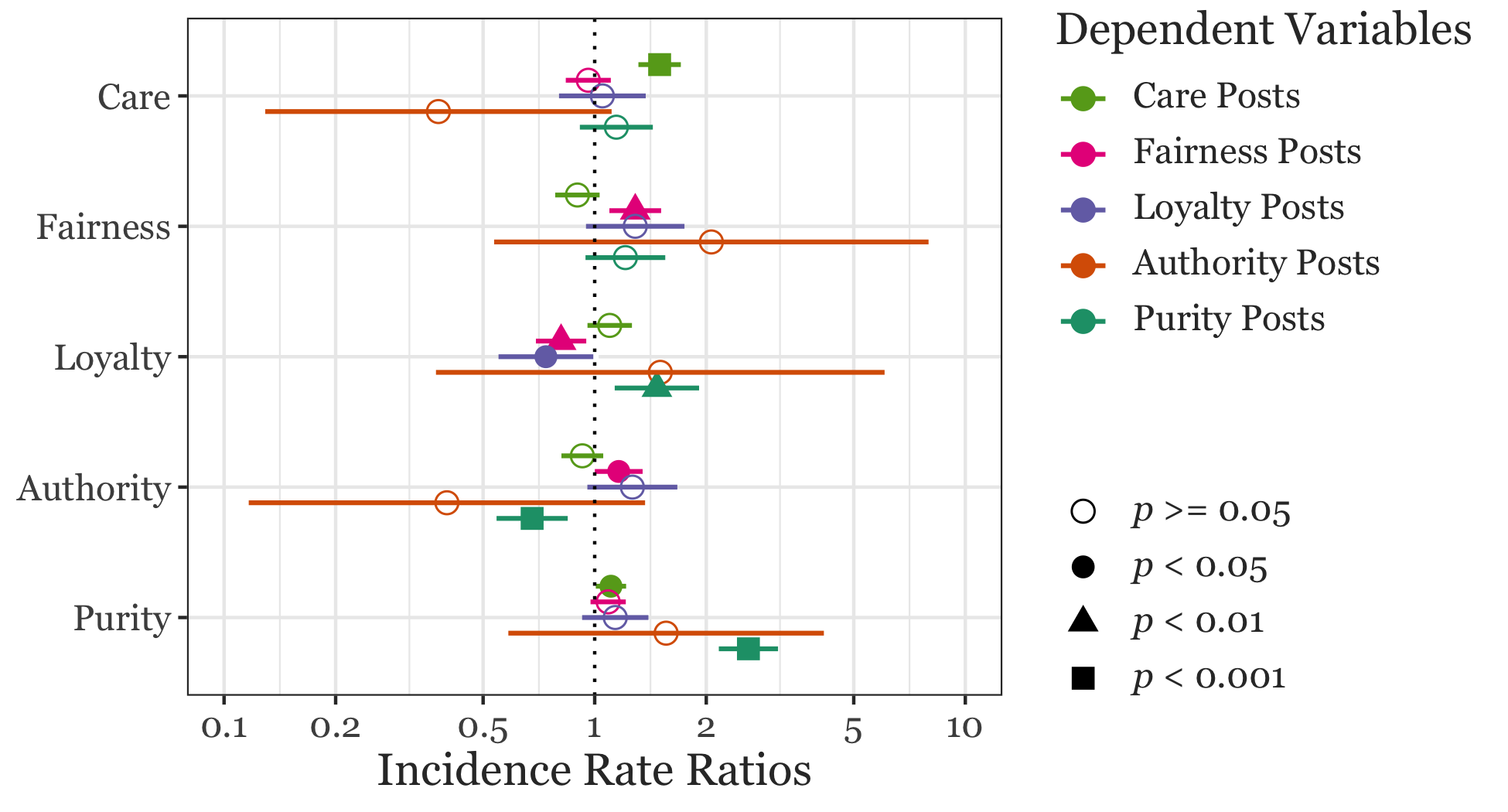


**Figure S10**

*Incidence Rate Ratios for Poisson Models Predicting the Number of Posts Based on Age and Gender. Bars are 95% Confidence Intervals*


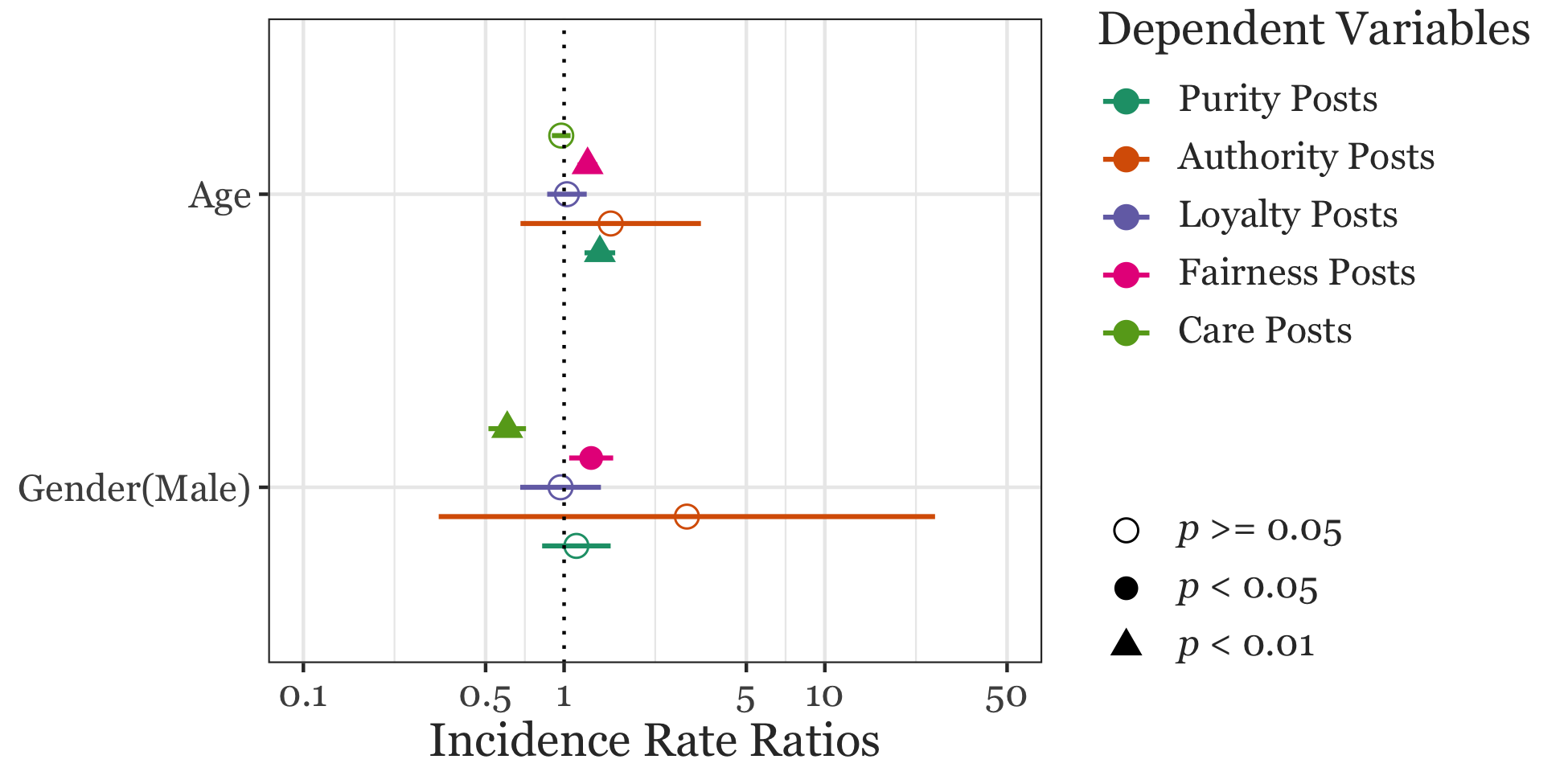


Lastly, participants’ gender and standardized age were modeled as predictors of the frequency of each moral category posted on Facebook by each participant. Poisson regressions were again used (10 regressions, one each per moral category), offset by the log-transform of the number of posts. The likelihood ratios are visualized in Figure S10. In terms of model coefficients, women had a higher rate of care posts (β_Male_ = -0.502, *SE* = 0.085, *p* < 0.001) and men had higher rates of cheating posts (β = 0.608, *SE* = 0.082, *p* < 0.001), fairness posts (β = 0.239, *SE* = 0.099, *p* = 0.016), and harm posts (β = 0.500, *SE* = 0.151, *p* < 0.001). Age was also related to the frequency of moral Facebook posts: a 1 SD increase in participants’ age predicted higher rates of cheating (β = 0.148, *SE* = 0.035, *p* < 0.001), fairness (β = 0.207, *SE* = 0.045, *p* < 0.001), purity (β = 0.316, *SE* = 0.069, *p* < 0.001), and subversion (β = 0.444, *SE* = 0.131, *p* < 0.001) posts. Overall, the rate of moral posting increased with age, and men had a higher rate of moral posts than women with the notable exception of care posts.

**References**

Graham, J., Nosek, B. A., Haidt, J., Iyer, R., Koleva, S., & Ditto, P. H. (2011). Mapping the moral domain. *Journal of Personality and Social Psychology*, *101*(2), 366.

Mair, P., & Wilcox, R. (2020). Robust statistical methods in R using the WRS2 package. *Behavior Research Methods*, *52*(2), 464–488.
